# Rates of positive vs negative studies in the spine literature

**DOI:** 10.1016/j.inpm.2024.100423

**Published:** 2024-07-17

**Authors:** Samantha Levin, Joshua Levin

**Affiliations:** a Mountain View High School in Mountain View, CA, USA; b Department of Orthopaedic Surgery, Stanford University, 450 Broadway St., Pavilion C, 4th Floor, MC 6342, Redwood City, CA, 94063, USA; c Department of Neurosurgery, Stanford University, Palo Alto, CA, USA

**Keywords:** Low back pain, Neck pain, Positive studies, Negative studies

## Abstract

**Background:**

Accuracy in the interpretation of data, and publication of studies regardless of outcomes are vital to the development of the scientific literature.

**Objective:**

To determine the proportion of studies in the spine literature that report positive results.

**Study design:**

Review article of studies published in nine major spine, pain, and physical medicine and rehabilitation (PM&R) journals from January 1, 2018–December 31, 2022.

**Patient sample:**

Not applicable.

**Methods:**

Articles that reported on pain and/or function from 2018 to 2022 in nine major journals were reviewed by two independent evaluators. The articles were graded as either positive or negative based on the authors’ own conclusions about their work.

**Results:**

Overall, 91 % [95 % CI 88–94 %] of all articles were reported to have positive results. No significant differences were found between the broad categories of spine, pain, and PM&R journals. When comparing different categories of treatments, there were lower rates of positive results from medication/supplement studies (54 % [95 % CI 27–81 %]) compared to studies of spine injections/interventions (95 % [95 % CI 91–99 %]) and those of surgery (100 % [95 % CI 96–100 %]), and a lower rate of positive results from studies on physical treatments (85 % [95 % CI 75–95 %]) compared to those of surgery (100 % [95 % CI 96–100 %]). Studies with placebo controls were less likely to report positive results (60 % [95 % CI 44–76 %]) compared to those that did not use placebo controls (96 % [95 % CI 94–98 %]).

**Conclusions:**

Despite the vast majority of studies in the spine literature concluding positive results, the high disease prevalence of spine conditions and the enormous burden on the healthcare system remain.

## 1 Introduction

Low back pain affects over 619 million people globally and is the number one cause of disability throughout the world [1], and over 288 million people are affected by neck pain [2]. In the United States, neck and low back pain account for the highest amount of health care spending dollars with over $134 billion spent in 2016, representing a greater healthcare spend than diabetes, ischemic heart disease, skin disease, and hypertension [3].

In the medical literature, studies with negative results are less likely to be published than those with positive results [4,5]. Unpublished negative results lead to improper conclusions from meta-analyses, and therefore improper interpretation of the literature [6]. In orthopedic research, the acceptance rate of submitted articles has been shown not to differ between those with positive vs non-positive results, demonstrating that the higher publication rate of positive studies (73 % in this study) was due to a higher rate of submitted articles with positive results [7].

The purpose of this study was to evaluate the rates of studies on spine conditions that report positive versus negative results between 2018 and 2023.

## 2 Methods

Two evaluators independently reviewed all articles on the treatment of pain and/or function from January 2018–December 2022 from nine major journals (three spine, three pain, and three PM&R journals). The journals included were *The Spine Journal*, *Spine*, *European Spine Journal*, *Pain Medicine*, *Pain, Pain Physician*, *PM&R*, *Archives of PM&R*, and *The American Journal of PM&R* (see below for an explanation of journal selection). Each article was evaluated to determine the rates of studies that reported positive results. Inclusion criteria were original research articles (prospective or retrospective) that reported on outcomes related to pain and/or function for the treatment of any non-fracture, non-metastatic, non-infectious spine condition (cervical spine, thoracic spine, lumbar spine, sacrum/sacroiliac joint) in humans ≥18 years old. Exclusion criteria were studies that did not specifically evaluate a spine condition (i.e. chronic pain, musculoskeletal pain), review articles/meta-analyses, case reports or case series of less than 10 patients, letters to the editor, expert consensus/clinical guidelines, studies reporting on radiographic outcomes without pain or functional outcomes, and cost benefit analyses. If a study compared two or more treatments and the authors did not clearly frame their findings as either positive or negative (i.e. if the results were reported as equal and this was not framed as positive or negative results), the study was excluded.

Journal selection for our study began by generating two separate lists using scores from Impact Factor and Scopus for four separate medical specialties (spine, pain, PM&R, and anesthesiology), with the goal of selecting three clinical medicine journals from each category that included a minimum of five articles that met the inclusion criteria over the five year data collection period. After developing the lists and evaluating the journals, only the spine category was successful in finding three journals that included at least five articles that met the inclusion criteria over the five-year time period. Table 1 shows the journals with the highest 2022 scores from Impact Factor and from Scopus for each category. Since this method of journal selection was not successful, a new method was developed – expert opinion.

**Table 1:** Top five Impact Factor and Scopus journals by category (2022 Impact Factor and Scopus scores in parentheses.).

Impact Factor	Scopus
**Spine journals**	**Spine journals**
The Spine Journal (4.5)	The Spine Journal (7.5)
Joint Bone Spine (4.2)	JOR Spine (6.1)
JOR Spine (3.7)	Spine (5.7)
Neurospine (3.2)	Journal of Neurosurgery Spine (5.4)
Spine (3.0)	Global Spine Journal (5.0)

**Pain journals**	**Pain journals**
Journal of Headache and Pain (7.4)	Pain (12.5)
Pain (7.4)	Journal of Headache and Pain (11.6)
Anesthesia Critical Care and Pain Medicine (5.5)	Headache (8.9)
Pain Reports (4.8)	Journal of Pain (7.9)
Journal of Pain and Symptom Management (4.7)	Regional Anesthesia and Pain Medicine (7.6)

**PM&R journals**	**PM&R journals**
Journal of Physiotherapy (12.1)	Annals of PM&R (7.3)
Journal of Orthopaedic & Sports Physical Therapy (6.1)	European Journal of Physical and Rehabilitation Medicine (7.3)
IEEE Transactions on Neural Systems and Rehabilitation Engineering (4.9)	Physical and Engineering Sciences and Medicine (7.0)
Disability and Health Journal (4.5)	Physics in Medicine and Biology (6.9)
Archives of PM&R (4.3)	Archives of PM&R (6.2)

**Anesthesia journals**	**Anesthesia journals**
Anesthesia (10.7)	Anesthesiology (9.6)
British Journal of Anesthesia (9.8)	Anesthesia and Analgesia (8.7)
Pain (7.4)	Canadian Journal of Anesthesia (8.2)
Journal of Clinical Anesthesia (6.7)	Korean Journal of Anesthesiology (7.8)
Anesthesia and Analgesia (5.9)	Regional Anesthesia and Pain Medicine (7.6)

A brief survey was then sent to ten current or former members of the Board of Directors from the International Pain and Spine Intervention Society (IPSIS), asking them to rank their top three journals from each of the four categories. They were not given information about the study. They were simply asked to provide their top three choices for journals that report on pain and/or functional outcomes for spine conditions. Nine of the ten experts replied, so one additional survey was sent to another expert in order to reach the pre-set goal of obtaining recommendations from ten experts. Each expert's top choice was given three points, the second choice two points, and the third choice one point. Using this scoring system, a list of journals with the top three scores from each category was developed. The same requirement for at least five articles over the five-year period was used to develop the final list. This final list consisted of three spine journals, three pain journals, and three PM&R journals. None of the anesthesia journals had at least five articles that met criteria over the five-year time period, and therefore none were included in the final list (Table 2).

**Table 2:** List of the top three journals by category generated by expert opinion. Only journals that had at least five articles that met inclusion criteria over the five-year time period are marked by an * and were included in the final list of journals.

**Spine journals**
*The Spine Journal
*Spine
*European Spine Journal

**Pain journals**
*Pain Medicine
*Pain
*Pain Physician

**PM&R journals**
*PM&R
*Archives of PM&R
*American Journal of PM&R

**Anesthesia journals**
Regional Anesthesia & Pain Medicine
Anesthesiology
Anesthesia & Analgesia

The senior author reviewed the journals to determine which articles met the inclusion criteria. Then, the two evaluators performed their analyses independently. For each journal, the evaluators visited the journal's website and read the abstract from each article. If the abstract did not provide sufficient information, the evaluators then read the article. A positive study was defined as one in which the authors of that study concluded that the results of their study showed a benefit in the treatment of pain and/or function. If an article studied one treatment as the focus of the study, and if that treatment was not better than a different treatment (active control or placebo), the article was considered to be a negative study. However, if both treatments were framed as being successful, it was counted as a positive study. See Table 3 for definitions of positive and negative studies.

**Table 3:** Definition of positive and negative studies.

Positive Study:	*Treatment being studied is beneficial (no control group)
	*Treatment being studied is better than placebo
	*Treatment being studied is better than a second treatment
	*Treatment being studied has similar results to another treatment, and both are beneficial
Negative Study:	*Treatment being studied is not beneficial (no control group)
	*Treatment being studied is not better than placebo
	*Treatment being studied is not better than a second treatment
	*Treatment being studied has similar results to another treatment, and neither is beneficial

Proportions of positive studies were determined for each journal separately and for each category of journals (spine, pain, and PM&R). Additionally, treatments were divided into the following six broad categories: physical treatments (i.e. physical therapy, chiropractic treatment, exercise, modalities), spine injections and other interventional treatments (such as percutaneous radiofrequency ablations), medications/supplements, psychology/behavioral health treatments, surgery, and alternative treatments (i.e. acupuncture/acupressure, bright light therapy, transcranial stimulation). Results were evaluated based on these treatment categories.

The two evaluators did not review the original data from the studies that were evaluated, and they did not interpret the findings of the studies themselves. Distinctions were not made on primary vs secondary outcomes, or primary vs secondary timepoints. Instead, the purpose of our study was simply to determine the frequency in which authors presented the conclusions of their work in a positive manner (i.e., “our treatment helps”). This is the reason why studies that did not clearly frame their results as positive or negative were excluded from our analysis. Since this was a unique outcome measure that could involve subjective interpretation, two evaluators were used in our study, and inter-rater reliability was calculated. Disagreements were resolved by consensus.

## 3 Statistical analysis

Inter-rater reliability was assessed by calculating the simple kappa statistic with a 95 % confidence interval (CI). The proportions of studies that were graded as positive were calculated along with Wald 95 % confidence intervals. Similar calculations were performed for each journal individually, for each category of journals, and for each category of treatment. Additionally, similar calculations were performed for the subset of studies that were placebo-controlled.

## 4 Results

A total of 280 articles met the inclusion criteria and were evaluated. The inter-rater reliability analysis showed strong agreement with a kappa statistic of 0.84 [95 % CI 0.74–0.95]. A total of seven disagreements occurred between the two evaluators, and consensus was reached using the senior author's results for five of the seven disagreements.

Of the 280 studies, 256 were positive (91 % [95 % CI 88–94 %]). The only journal whose rate of positive studies differed from other journals was *Pain* (60 % [95 % CI 39–81 %]) which had a lower rate of positive studies compared to *The Spine Journal* (100 % [95 % CI 85–100 %], *Pain Physician* (98 % [95 % CI 95–100 %]), and *European Spine Journal* (97 % [95 % CI 93–100 %]). See Table 4. No significant differences were present when comparing the three categories of journals (spine, pain, and PM&R). See Table 5.

**Table 4:** Rates of positive studies by individual journals.

All Journals	91 % (256/280) [95 % CI 88–94 %]

*The Spine Journal*	100 % (23/23) [95 % CI 85–100 %]
*Pain Physician*	98 % (64/65) [95 % CI 95–100 %]
*European Spine Journal*	97 % (59/61) [95 % CI 93–100 %]
*Archives of PM&R*	92 % (12/13) [95 % CI 77–100 %]
*Spine*	90 % (37/41) [95 % CI 81–99 %]
*Pain Medicine*	90 % (37/41) [95 % CI 81–99 %]
*PM&R*	83 % (5/6) [95 % CI 53–100 %]
*American Journal of PM&R*	70 % (7/10) [95 % CI 42–98 %]
*Pain*	60 % (12/20) [95 % CI 39–81 %]

**Table 5:** Rates of positive studies by journal categories.

Combined Spine Journals	95 % (119/125) [95 % CI 91–99 %]
Combined Pain Journals	90 % (113/126) [95 % CI 85–95 %]
Combined PM&R Journals	83 % (24/29) [95 % CI 69–97 %]

Of the six broad treatment categories, 73 % of the 256 positive articles were studies of invasive treatments (surgery or injection/interventions), 17 % were studies on physical treatments, 4 % were studies on psychological/behavioral treatments, 3.5 % were studies on alternative treatments, and 2.7 % were studies on medications/supplements. Medication/supplement treatments showed a lower rate of positive studies (54 % [95 % CI 27–81 %]) compared to surgery (100 % [95 % CI 96–100 %]) and to injection/intervention treatments (95 % [95 % CI 91–99 %]), and physical treatments showed a lower rate of positive studies (85 % [95 % CI 75–95 %]) compared to surgery (100 % [95 % CI 96–100 %]). See Table 6.

**Table 6:** Rates of positive studies by treatment category.

Surgery	100 % (90/90) [95 % CI 96–100 %]
Injections/Interventions	95 % (96/101) [95 % CI 91–99 %]
Alternative treatments	90 % (9/10) [95 % CI 71–100 %]
Physical treatments	85 % (44/52) [95 % CI 75–95 %]
Psychology/Behavioral treatments	79 % (11/14) [95 % CI 58–100 %]
Medication/Supplement	54 % (7/13) [95 % CI 27–81 %]

Thirty-five of the 280 studies were placebo-controlled studies. Of these, 60 % [95 % CI 44–76 %] were positive, and this rate was statistically lower than the rate of positive studies in those that did not use placebo controls (96 % [95 % CI 94–98 %]). Of the placebo-controlled studies, there were no significant differences in the rates of positive studies by journal categories or by treatment categories, however these comparisons were limited by small sample sizes. See Table 7.

**Table 7:** Rates of positive studies in placebo-controlled studies.

Overall	60 % (21/35) [95 % CI 44–76 %]

*Journal categories*:	
Combined Spine Journals	60 % (3/5) [95 % CI 17–100 %]
Combined Pain Journals	64 % (16/25) [95 % CI 45–83 %]
Combined PM&R Journals	40 % (2/5) [95 % CI 0–83 %]

*Treatment categories*:	
Surgery	0 % (0/0)
Injections/Interventions	64 % (9/14) [95 % CI 39–89 %]
Alternative treatments	83 % (5/6) [95 % CI 53–100 %]
Physical treatments	60 % (3/5) [95 % CI 17–100 %]
Psychology/Behavioral treatments	50 % (1/2) [95 % CI 0–100 %]
Medication/Supplement	38 % (3/8) [95 % CI 4–72 %]

## 5 Discussion

The vast majority of studies in the spine literature from 2018 to 2022 reported positive results. Given the high prevalence of spine conditions [1,2] and the enormous amount of healthcare dollars spent on them [3], one might expect that most studies would show that their treatments were ineffective. However, our study showed quite the opposite finding. Almost all studies (91 %) from the nine major journals that we reviewed reported positive results. The disconnect between these findings and the societal burden of these conditions is quite striking.

We investigated whether types of journals or types of treatments had a significant effect on the rates of positive studies, and we mostly showed no differences. Spine, pain, and PM&R journals showed similar rates of positive studies. One finding that was seen, was that invasive treatments were more likely to be framed as having positive results than some conservative treatments – a finding that was not unexpected.

Another interesting, and also not unexpected finding was that placebo-controlled studies were less likely to report positive results than studies that did not use placebos. While the rate of placebo-controlled studies that reported positive results (60 %) was lower than the rate for studies that did not use placebos (96 %), this rate is still higher than might be expected for studies on the treatment of spine conditions. This demonstrates the importance of critical interpretation of studies, even when placebo controls are used. Lastly, the fact that virtually all non-placebo-controlled studies (96 %) reported positive results is concerning and demonstrates the importance and need for higher quality literature.

Studies with negative results are frequently not published due to a variety of reasons including the inherent bias of humans to focus on positive results, authors favoring to submit studies with positive results, journals favoring studies with positive results, fear of the perception that may accompany negative results, time constraints of investigators who tend to focus on positive findings, pressure to publish, and financial conflicts of interest [[8], [9], [10], [11]]. Another reason for the lack of negative studies is a simple misunderstanding of how to interpret results. As Teixeira da Silva stated, “there might be a wealth of negative results with very positive messages”(10). When comparing two treatments, if both result in “positive results”, the proper conclusion is *not* that both treatments “work”. On the contrary, unless a placebo control is used, the only conclusion that can accurately be drawn is that there is no difference between the two groups. Whether this means that both treatment arms were successful, or that both would be no better than placebo, cannot be determined. Therefore, results from such a study should not be definitively considered to be positive. Lastly, not only are positive studies more likely to be published, they are also more likely to be cited [12,13]. This can further exacerbate the publication bias against negative studies.

Our study has limitations. First, our journal selection was based on expert opinion. We attempted to use Impact Factor and Scopus to develop an objective list of journals, however most journals selected by this method did not meet our requirement for having at least five articles that met our inclusion criteria over the five-year period of our review. The reasons for this varied from the inclusion of basic science journals to the inclusion of journals that focus on non-spine conditions (such as headache journals) to the inclusion of journals that focus on studies that do not evaluate the treatment of pain and/or disability from spine conditions. Including these journals would not have produced a sufficient number of articles for our study. After evaluating five journals from each category by using this method, it was determined that we would not be able to go down the lists and review five years of articles from every journal in a systematic manner until we found three journals that met our requirement of having at least five articles that met our inclusion criteria. After switching to expert opinion, two thirds of the journals in our final list included at least 20 articles that met our inclusion criteria, and only one had fewer than 10 articles. None of the three journals with the most articles (*Pain Physician*, *Pain Medicine*, and *European Spine Journal*) were in the top five of the lists generated from Impact Factor and Scopus, and only four of the journals from our final list (*The Spine Journal*, *Spine*, *Pain*, and *Archives of PM&R*) were also in the top five journals from the lists that were generated by using Impact Factor and Scopus.

Second, our final evaluation only consisted of nine journals from three broad categories. Many other journals report on spine conditions, and our results cannot be assumed to be generalizable to journals that we did not include. We attempted to include journals from the field of anesthesiology, however none of the journals in our lists included enough studies that met our inclusion criteria. Additionally, journals from other fields of medicine, such as rheumatology and chiropractic, also report on outcomes from spine treatments, and could have different results than the journals that we reviewed.

Another limitation is that we relied on the authors' conclusions of their study results. We did not provide our own interpretation of whether a study should have been framed as having positive or negative results. However, this was done by design – the purpose of our study was to evaluate how often authors conclude that their work is beneficial. Our study was not designed as a critical review of the proper interpretation of the results from each study – it was merely a review of the authors’ conclusions.

Finally, our review was limited to the most recent five-year time frame, and therefore no conclusions can be drawn about studies from previous years. The fact that negative studies in the scientific literature gradually became less common from 1990 to 2007([14]), combined with our findings, suggests that the issue of publication bias against negative studies continues to worsen.

## 6 Conclusions

Given the high prevalence of spine conditions, affecting approximately 1 billion people worldwide, one would expect that many current and previous spine treatments are not successful. Yet, the large majority of the spine literature from 2018 to 2022 report positive results.

## Funding

This research did not receive any specific grant from funding agencies in the public, commercial, or not-for-profit sectors.

## Declaration of competing interest

The authors declare the following financial interests/personal relationships which may be considered as potential competing interests:

Joshua Levin reports a relationship with State Farm Insurance Companies that includes: consulting or advisory. Joshua Levin reports a relationship with Liberty Mutual Group Inc that includes: consulting or advisory. Joshua Levin reports a relationship with Allstate Insurance that includes: consulting or advisory. Joshua Levin reports a relationship with Hanover Insurance Group that includes: consulting or advisory. Joshua Levin reports a relationship with GEICO that includes: consulting or advisory. If there are other authors, they declare that they have no known competing financial interests or personal relationships that could have appeared to influence the work reported in this paper.
